# Editorial: Maternal dietary and lifestyle patterns with pregnancy, birth, and child health outcomes

**DOI:** 10.3389/fnut.2023.1266598

**Published:** 2023-10-17

**Authors:** Shan Xuan Lim, Jun Shi Lai, Ling-Wei Chen, Airu Chia

**Affiliations:** ^1^Saw Swee Hock School of Public Health, National University of Singapore and National University Health System, Singapore, Singapore; ^2^Singapore Institute for Clinical Sciences, Agency for Science, Technology and Research, Singapore, Singapore; ^3^Institute of Epidemiology and Preventive Medicine, College of Public Health, National Taiwan University, Taipei, Taiwan

**Keywords:** preconception exposures, pregnancy exposures, postpartum exposures, gestational weight gain, childhood obesity, maternal and child health, maternal diet, maternal lifestyle

Evidence from historical cohorts suggested that the children and grandchildren of women exposed to dietary alterations (e.g., famine) during pregnancy may experience increased risks of later health complications than their “control” counterparts (1). These findings drew attention to the importance of maternal pregnancy exposures and their implications on maternal/child health. Subsequently, mounting evidence revealed that maternal dietary, lifestyle (e.g., smoking, sedentary behaviors), and sociodemographic factors during the preconception, pregnancy and postpartum stages were associated with subsequent maternal and child outcomes (e.g., gestational diabetes, birth weight) among healthy, Caucasian women with access to health care (2, 3). However, there is insufficient evidence among non-Caucasian women and those of lower socioeconomic status (2) as well as paternal factors, despite their likely influence on both the mother and the child. The current literature highlighted the importance of micronutrients, adopting healthy diets, and optimizing various lifestyle behaviors to prevent adverse maternal and child health outcomes (4). Yet, few studies have examined the entirety of lifestyle behaviors. Addressing these research gaps will enable robust conclusions to be drawn and advance research in this field.

In this Research Topic, we collated nine articles featuring the latest research on maternal or paternal dietary, lifestyle and sociodemographic factors with subsequent maternal and child outcomes (5, 6). Notably, there is a good representation of study populations, with six studies from Asia- China (Chen et al., Li et al., Qin et al., Tao et al., Xie et al., Zhao et al.,), one study from Africa- Ethiopia (Ahmed et al.) and two studies from European countries- one involved a consortium of studies conducted in France, Ireland, and the Netherlands (Lecorguillé et al.) and one in Norway (Amberntsson et al.). Among these studies, one investigated preconception exposure (Li et al.), two investigated both preconception and pregnancy exposures (Lecorguillé et al., Xie et al.), four investigated maternal pregnancy exposures (Amberntsson et al., Chen et al., Qin et al., Tao et al.) and two investigated postpartum exposures (Ahmed et al., Zhao et al.).

Across preconception (Li et al.) and both preconception and pregnancy (Lecorguillé et al., Xie et al.) periods, maternal supplemental folate was associated with risk of gestational diabetes mellitus (Li et al.) and a combination of lifestyle factors (BMI, smoking, diet quality and sedentary behavior) was associated with higher risk of offspring overweight/obesity (Lecorguillé et al.). Additionally, maternal pre-pregnancy BMI and gestational weight gain were associated with sex-specific membership in BMI-z trajectories from birth to 5 years of age (Xie et al.). Future studies can consider investigating whether these trajectories would be associated with subsequent pubertal or disease-risk outcomes in children. Collectively, these three studies have certainly added to the limited literature on exposures during the preconception period, especially among non-Caucasian women (Li et al., Xie et al.). Notably, Lecorguillé et al. considered the synergistic effect of both maternal and paternal lifestyle factors that are valuable to support the initiation of family-based and multi-behavioral prevention strategies for childhood obesity.

Spanning the pregnancy period, Amberntsson et al., Chen et al., and Tao et al. demonstrated the associations between vitamin D intakes, serum levels of iron, magnesium, zinc, copper, and ferritin with maternal postpartum anemia and offspring birthweight outcomes. While these studies have identified several micronutrients associated with maternal and child health, results should be interpreted with caution as it is important to consider that nutrient bioavailability varies when interacting with other dietary constituents consumed in the diet (7). This limits our ability to directly translate the findings to dietary recommendations. As opposed to examining individual nutrients, the study by Qin et al. managed to circumvent these limitations by studying links between maternal dietary patterns and fetal intrauterine development. In particular, mothers who adhered to the “Snack and less eggs” low-protein pattern had increased risk of smaller head circumference for gestational age, which might implicate long-term health outcomes. This work by Qin et al. has the potential to inform the development of Chinese dietary guidelines for pregnant women to prevent adverse offspring health outcomes.

Spanning the postpartum period, Zhao et al. noted that Chinese women with a cesarean section birth tended to adhere less to the “Traditional” dietary pattern (characterized by high carbohydrates foods, fish and eggs) than those with vaginal delivery (Zhao et al.) among women with monthly household income of more than ¥9,000. The study also reported a positive correlation between cesarean section delivery and vegetable intakes only among those with tertiary education (Zhao et al.). Likewise, Ahmed et al. reported that lower maternal educational status and lower family income were correlated with stunting among 6–59 months old children to employed mothers in Ethiopia. While both of these studies are cross-sectional and causality cannot be inferred, they generated hypotheses for future studies and drew attention to the socio-demographic determinants for the health and wellbeing of maternal and child populations.

Finally, the studies included in this Research Topic have made valuable contributions to the evidence on parental factors and pregnancy, birth or childhood health outcomes. While single-nutrient studies have laid the foundation for dietary investigations, the approach of assessing overall diet and lifestyle is more comprehensive. Taken together, maintaining a healthy weight status, adopting a better-quality diet, reducing smoking exposure, and limiting sedentary lifestyles before and during pregnancy in both parents emerge as crucial factors in preventing adverse maternal and child health outcomes. Given the vast amount of information collected by the various cohorts featured in the nine articles, there remains many interesting hypotheses to explore that will advance research in this field. As these studies typically assessed exposures at a single time point, future studies may consider and examine changes (or trajectories) of diet, lifestyle, and sociodemographic exposures from preconception, pregnancy and postpartum periods. It may also be worth delving into the social determinants of maternal and child health such as community and family support, which could help with designing and implementing effective lifestyle interventions to optimize health and wellbeing.

## Author contributions

SXL: Conceptualization, Writing—original draft. JSL: Conceptualization, Supervision, Validation, Writing—review and editing. L-WC: Conceptualization, Supervision, Validation, Writing—review and editing. AC: Conceptualization, Supervision, Validation, Writing—review and editing.
